# Effects of exercise training on physical fitness of youth table tennis players: a systematic review

**DOI:** 10.3389/fphys.2026.1782585

**Published:** 2026-03-24

**Authors:** Yixuan Liu, Borhannudin Bin Abdullah, Hazizi Bin Abu Saad, Kehan Li, Changlong Fan

**Affiliations:** Department of Sports Studies, Faculty of Educational Studies, Universiti Putra Malaysia, Serdang, Malaysia

**Keywords:** agility, physical fitness, speed, strength, table tennis

## Abstract

**Background:**

Exercise training is essential for table tennis players, as identifying the most effective approach to enhance their physical fitness is crucial.

**Methods:**

Following the PRISMA guidelines, a total of 10 studies were included from 102 records identified through PubMed, EBSCOhost, Web of Science, Scopus, and Google Scholar (from inception to February 2026). Methodological quality, assessed using the PEDro scale, yielded a ranged from four to six, indicating acceptable research rigor and relatively reliable findings.

**Results:**

The findings indicate that strength (n = 9) and speed (n = 7) are the most frequently targeted attributes in training interventions, followed by agility (n = 5), flexibility (n = 5), and endurance (n = 4). The majority of studies support the effectiveness of exercise training in enhancing physical fitness among young table tennis players. However, inconsistencies remain regarding the extent of training-induced improvements in strength, speed, and endurance.

**Conclusion:**

This review demonstrates that exercise training programs are most effective for the fitness of youth table tennis players. Such training methods can improve athletes’ speed, strength, endurance, agility, flexibility showing clear advantages over traditional training approaches. However, current studies are limited by heterogeneity in training protocols and age groups, and a lack of long-term follow-up data. Future studies should employ standardized protocols, and long-term follow-up to determine optimal training strategies for youth table tennis athletes.

**Systematic Review Registration:**

https://inplasy.com, identififier INPLASY202530064.

## 1 Introduction

Table tennis is a high-speed net-based sport that requires the integrated coordination of technical precision, tactical adaptability, psychological regulation, and physical conditioning to achieve high-level performance (Kondrič et al., 2013; Fuchs et al., 2018; Pradas de la Fuente et al., 2013; Behaein et al., 2025; Chen Z. et al., 2025; Zagatto et al., 2010). The sport is characterized by explosive stroke production, rapid reactions to unpredictable ball trajectories, and frequent multidirectional movements performed within a confined playing space (Bańkosz et al., 2020; Nikolic et al., 2014; Faber et al., 2016; Liu et al., 2024). Its intermittent match structure involves repeated high-intensity efforts interspersed with brief recovery periods, imposing substantial neuromuscular and metabolic demands on both anaerobic and aerobic energy systems. These physiological requirements indicate that physical fitness is not merely complementary to technical skill, but rather constitutes the foundational capacity that enables effective stroke execution, movement efficiency, and sustained competitive performance (Picabea et al., 2021; Pradas et al., 2022).

In youth athletes, physical fitness development is particularly significant due to ongoing growth, neuromuscular maturation, and long-term athletic development considerations (Bańkosz et al., 2020; He et al., 2021). Appropriate training stimuli during adolescence may substantially influence strength acquisition, coordination efficiency, and energy system development, thereby shaping future performance potential. Match analysis data from youth competitions further indicate that stroke velocity, change-of-direction ability, and acceleration patterns are critical determinants of successful performance outcomes, with players required to repeatedly generate force and speed while maintaining technical accuracy under fatigue conditions (Faber et al., 2015; Dong et al., 2025; Chen and Wang, 2022; Quan and Zhao, 2023). Consequently, structured physical conditioning plays a critical role in supporting performance stability during high-tempo rallies and extended match play in developing athletes.

Physical fitness in youth table tennis is multidimensional, encompassing interrelated components that collectively support sport-specific performance (Kondrič et al., 2013; He et al., 2021). Speed enables rapid response to dynamic rallies; muscular strength underpins force production and change-of-direction ability; endurance sustains repeated high-intensity efforts; flexibility facilitates efficient stroke mechanics and dynamic footwork; and agility supports rapid repositioning around the table (Kondrič et al., 2013; Pradas de la Fuente et al., 2013; Picabea et al., 2021; Pradas et al., 2022; Pradas et al., 2021a). Evidence from other youth racket sports, including badminton, squash, and tennis, suggests that structured interventions such as high-intensity interval training, resistance-based strength programs, plyometric training, and agility-focused drills can effectively enhance sport-specific physical capacities (Pradas et al., 2021a; Liu et al., 2024). These programs commonly target multiple physiological domains, including muscular strength and power, acceleration and speed, aerobic and anaerobic endurance, and movement coordination. While caution is necessary when extrapolating findings across sports due to discipline-specific differences, such evidence provides a useful conceptual framework for evaluating training effects in youth table tennis. However, despite the recognized importance of physical conditioning in adult table tennis, research specifically examining structured training interventions in youth table tennis players remains limited, with existing literature often reporting heterogeneous protocols and isolated outcomes that limit comprehensive interpretation of training effectiveness (Pradas et al., 2010; Pradas et al., 2021b; Zagatto et al., 2017).

To date, no systematic review has comprehensively synthesized the effects of structured physical training interventions specifically on youth table tennis players. Current evidence is fragmented across different performance domains and study designs, making it difficult to draw integrated conclusions regarding optimal training strategies. A structured synthesis of available research is therefore warranted to clarify which physical fitness components are most responsive to targeted interventions and how improvements may translate into sport-specific performance enhancement in youth athletes. Therefore, the present study aims to systematically examine the effects of exercise training on the physical fitness of youth table tennis players.

## 2 Materials and methods

### 2.1 Protocol and registration

This systematic review was conducted in accordance with the Preferred Reporting Items for Systematic Reviews and Meta-Analyses (PRISMA) guidelines (Sarkis-Onofre et al., 2021). The review protocol was registered prospectively with the INPLASY database on March 15, 2025 (registration number: INPLASY202530064).

### 2.2 Search strategy

A comprehensive literature search was performed across four major electronic databases: PubMed, EBSCOhost, Web of Science, and Scopus. To enhance the scope of the review, gray literature and additional sources identified through Google Scholar were also considered. The search covered publications from the inception of each database to February 19, 2026.

The search strategy was tailored to each database. In PubMed, a combination of Medical Subject Headings (MeSH) terms and free-text keywords was used. The primary MeSH terms included “Table Tennis” and “Physical Fitness,” while free-text keywords such as “strength,” “speed,” and “agility” were applied to capture related concepts. Boolean operators (AND, OR) were employed to combine search terms. An example search string was: (“table tennis” OR “ping pong”) AND (“physical fitness” OR strength OR speed OR agility). Equivalent keyword combinations were adapted for EBSCOhost, Web of Science, and Scopus using free-text terms in accordance with the specific syntax of each database.

### 2.3 Eligibility criteria

This study adopted the PICOS framework as the basis for defining inclusion criteria. PICOS outlines five core elements: Population, Intervention, Comparison, Outcomes, and Study design, which serve to structure the research question and refine the scope of a systematic review. This framework facilitated a clear and consistent approach to literature selection and eligibility assessment (Table 1).

**TABLE 1:** Inclusion criteria based on PICOS.

PICOS	Detail
Population	Youth table tennis players aged 15–24
Intervention	Any type of exercise training
Comparison	Single-group trials, two-group trials and multiple
Outcome	Physical fitness components (speed, strength, endurance, flexibility, agility)
Study designs	RCTs

The inclusion criteria for this review were defined as follows: (P) Population: Participants were required to be healthy youth table tennis players of either sex. Conceptually, the term youth was defined in accordance with the World Health Organization, which classifies youth as individuals aged 15–24 years (Mansfie et al., 2018). This operational definition was adopted to ensure sufficient evidence coverage while maintaining conceptual consistency with youth-focused training and performance development.

1. Intervention: Studies investigating exercise training interventions were included, encompassing both single-modality and multi-component programs. These interventions covered a broad range of training types, such as strength and power training, speed and agility drills, aerobic or anaerobic conditioning, and combined physical training approaches. (C) Comparison: Studies included comparisons of different training interventions, control groups, or pre- and post-intervention outcomes within a single group. (O) Outcome: The study must evaluate the effects of exercise training, such as speed strength, endurance, flexibility or agility, on youth table tennis players. (S) Study design: Studies must be either randomized controlled trials (RCTs). Given the inclusion of studies with different methodological designs, variations in levels of evidence were addressed through qualitative comparison during narrative synthesis, rather than statistical pooling. No subgroup analysis was conducted due to heterogeneity in study designs and outcome measures.

### 2.4 Study selection

After two independent authors selected articles that met the inclusion criteria, this review employed an EndNote citation management system to identify and remove duplicates. The titles and abstracts of the papers were assessed by Liu and Borhannudin to determine their suitability for inclusion in this study. In cases where the two authors disagreed on the selection of an article, a third author conducted a comprehensive analysis of the full article to make the final decision.

### 2.5 Data extraction and quality assessment

Population characteristics included sample size and group allocation (experimental and control groups), number of participants, height and body mass, age, sex distribution, and training level. Where available, participants’ training background and competitive level (e.g., elite, sub-elite, or amateur) were also extracted, as these factors are important for interpreting physiological adaptations to exercise training. Intervention details described the type of training program (e.g., special physical training, flexibility training, or traditional conditioning), training frequency, session duration, total intervention period, and specific training contents or measurement variables. Outcomes comprised indicators of physical fitness such as strength, speed, endurance, flexibility, agility, and sport-specific performance measures.

To ensure a rigorous appraisal of trial quality, the Physiotherapy Evidence Database (PEDro) scale was employed (Shiwa et al., 2011). The PEDro scale is a validated instrument widely used in physical therapy and allied health sciences to assess methodological quality and internal validity (Maher et al., 2003). It consists of 11 criteria that evaluate core aspects of study design, including randomization, blinding, and statistical reporting. Given the nature of exercise training interventions, blinding of participants and trainers was generally not feasible. As a result, most included studies did not meet the PEDro blinding criteria, and this inherent limitation was considered when interpreting methodological quality scores. Each item is scored as either present or absent, resulting in a total score ranging from 0 to 10, where higher scores reflect stronger methodological rigor and lower risk of bias.

According to the included literature, studies scoring between 8 and 10 were categorized as having excellent methodological quality. Those with scores between five and seven were classified as high quality. Scores ranging from three to four were considered to indicate moderate methodological quality. Studies scoring below three were regarded as lacking sufficient rigor and were excluded from further synthesis (Sherrin et al., 2000).

## 3 Result

### 3.1 Study selection

**FIGURE 1:**
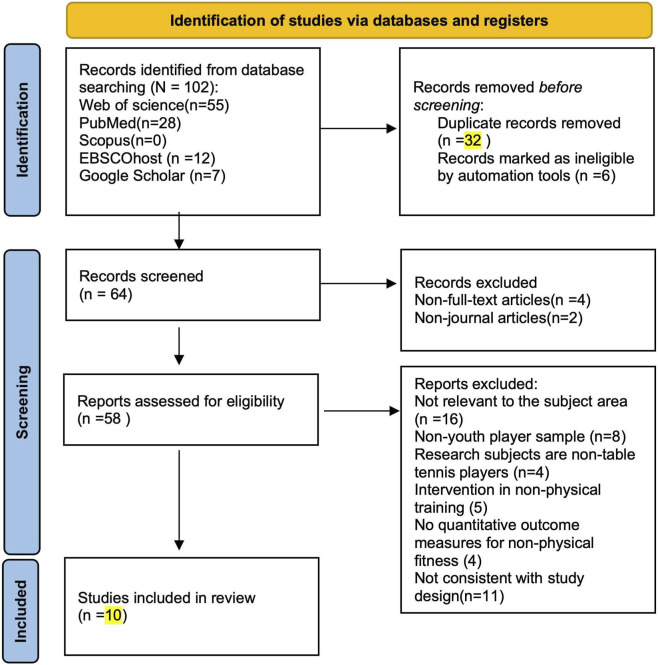
The preferred reporting items for systematic reviews and meta-analyses (PRISMA) flow diagram of the literature selection process.

A total of 102 records were initially identified through database searches, including 55 from Web of Science, 28 from PubMed, 12 from EBSCOhost, and seven from Google Scholar, with no records retrieved from Scopus. In addition, reference lists of relevant studies and grey literature sources (e.g., theses, dissertations, and conference papers) were screened to ensure comprehensive coverage. Prior to screening, 32 duplicate records and six automatically excluded entries were removed using EndNote reference management software. During the initial screening phase, 6 records were excluded, including four non–full-text items and two non-journal publications (e.g., book chapters or conference abstracts). Subsequently, 58 full-text articles were assessed for eligibility. Among them, 16 were excluded for being unrelated to the subject area, eight for involving non-youth player samples, four for using non–table tennis participants, five for including interventions unrelated to physical training, four for lacking quantitative physical fitness outcomes, and 11 for inconsistent study designs (e.g., absence of pre-post tests or control groups). Ultimately, 10 studies met the inclusion criteria and were included in the qualitative synthesis. The detailed screening process and reasons for exclusion are detailed in the PRISMA flow chart (Figure 1).

### 3.2 Study quality assessment

As shown in Table 2, the methodological quality of the included studies ranged from four to six on the PEDro scale. Five studies achieved a score of 6, three studies scored 5 (Moseley et al., 2020; Hao, 2023; Meng, 2023), and two studies scored 4 (Dehua, 2023). Overall, the majority of trials demonstrated adequate methodological rigor in aspects such as randomization, baseline comparability, and statistical reporting. However, none of the studies sufficiently addressed allocation concealment or incorporated blinding procedures for participants, therapists, or assessors. This omission reflects the practical constraints frequently encountered in exercise-based research, where masking of intervention groups remains inherently challenging. Nevertheless, the widespread use of intention-to-treat analysis and the presence of statistically robust between-group comparisons lend credibility to the reported comparative outcomes. The consistent scoring across studies not only reflects a satisfactory standard of methodological execution but also highlights domain-specific limitations that continue to shape research practices in sports science interventions.

**TABLE 2:** Methodological quality assessment.

Study	Eligibility criteria	Random allocation	Allocation concealment	Baseline comparability	Blind subjects	Blind therapist	Blind assessor	Follow up	Intention to treat analysis	Between group comparisons	Point measure and variability	Total PEDro score
Zhang (2022)	1	1	0	1	0	0	0	1	0	1	1	5
Wang and Zhou (2023)	1	1	0	1	0	0	0	1	1	1	1	6
Dehua (2023)	1	1	0	1	0	0	0	1	0	0	1	4
Meng (2023)	1	1	0	1	0	0	0	1	1	0	1	5
Hao (2023)	1	1	0	1	0	0	0	1	1	0	1	5
Xiong et al. (2022)	1	1	0	1	0	0	0	1	1	1	1	6
Chen Q. et al. (2025)	1	1	0	1	0	0	0	1	1	1	1	6
Zhu et al. (2024)	1	1	0	1	0	0	0	1	1	1	1	6
Wu et al. (2023)	1	1	0	1	0	0	0	1	1	1	1	4
Dong et al. (2025)	1	1	0	1	0	0	0	1	1	1	1	6

### 3.3 Participant characteristics

Table 3 summarizes the key characteristics of the 10 studies meeting the inclusion criteria. (1) Sample size: A total of 266 participants were included across the 10 studies, with sample sizes ranging from 12 (Zhang, 2022) to 40 (Hao, 2023). (2) Gender: Three studies targeted only male participants (Wang and Zhou, 2023; Zhang, 2022; Chen Q. et al., 2025), two studies focused solely on females (Xiong et al., 2022; Zhu et al., 2024), one study included both male and female participants (Wu et al., 2023), and four studies did not specify gender (Hao, 2023; Dehua, 2023; Meng, 2023; Chen Q. et al., 2025). (3) Age: Participants’ ages ranged from 15 to 24 years. The median age was 19.3 years, with a mean of 19.79 years (4) Training time: Three studies reported the participants’ training duration, which ranged from 5 to 13 years (Dong et al., 2025; Zhu et al., 2024; Zhang, 2022); the remaining seven studies did not report this information (Hao, 2023; Wang and Zhou, 2023; Xiong et al., 2022; Dehua, 2023; Meng, 2023; Chen Q. et al., 2025; Wu et al., 2023). (5) Athlete level: One study was conducted on provincial-level junior table tennis players (Chen Q. et al., 2025); seven studies involved university or college-level table tennis players (Dong et al., 2025; Hao, 2023; Wang and Zhou, 2023; Zhang, 2022; Dehua, 2023; Meng, 2023; Wu et al., 2023); one study included national-level or elite athletes (Xiong et al., 2022); and one study did not specify the athlete level (Zhu et al., 2024).

**TABLE 3:** Characteristics of included studies.

Study	Design	Type of athletes	Population characteristics	Interventions			Outcomes
				Exercises method in detailed	Group	Measures index	
Zhang (2022)	RCT	Table tennis players from the Institute of physical Education	E.G., = 6, CG = 6 Sex = male Age: 21.46 ± 2.3 (YR) Height:175 ± 6 Weight: 175.18 ± 3.14 Level: University table tennis player TT:5–8 (YR)	Freq.: 2 times/week, time: NR Length: 9 weeks	E.G.,:Progressive strength training CG: Traditional training	Shoulder joint flexion peak torque 60°/s Bending moment 450°/s Peak extension torque 60°/s Peak flexion torque of elbow joint 60°/s Bending moment 450°/s Peak extension torque 450°/s Peak extension torque of wrist joint 60°/s Peak extension torque 450°/s Bending moment 60°/s Bending moment 450°/s	Peak flexion torque of elbow joint 60°/s↑ Bending moment 450°/s↑ Peak extension torque 450°/s↑ Peak extension torque of wrist joint 60°/s↑ Peak extension torque 450°/s↑
Wang and Zhou (2023)	RCT	Chinese college young table tennis players	E.G., = 10, CG = 10 Sex = male Age: 20 (YR) Height:175.39 ± 1.83(CM) Weight: 73–78 (KG) Level: For students in professional sports schools TT:NR	Freq.: NR times/week, time:NR Length: 8 weeks	E.G.,:Lower limb flexibility training (static + dynamic CG: Traditional training	Strength: 1RM squat, CMJ jump height, fixed-point swing speed Speed: 30-M run Endurance: Number of squats in 1 min Flexibility: seated forward bend distance, hip flexion/extension angle, knee flexion angle, ankle flexion/dorsiflexion angle Agility: Cross quadrant jump, number of push-block side slides	1RM squat↑ CMJ jump height↑ 1 min squat times↑ Sit-and-reach↑ Hip flexion↑ Ankle dorsiflexion↑ Fixed-point swing speed↑ Push-and-slide times↑
Dehua (2023)	RCT	College young table tennis players	E.G., = 10, CG = 10 Sex = NR Age: 20.07 ± 1.20 (YR) Height:179.37 ± 4.63 (CM) Weight: 71.28 ± 11.31 (KG) Level: University table tennis player TT:NR	Freq.: 3 times/week, time:NR Length: 8 weeks	E.G.,: Upper limb strength resistance training CG: NR	Strength: Grip strength (kg): Handheld dynamometer test Endurance Plank support time (minutes)	Grip strength↑ Plank support time↑
Meng (2023)	RCT	Sport college young table tennis players	E.G., = 15, CG = 15 Sex = NR Age:19.298 ± 0.708 (YR) Height:172.833 ± 7.133 (CM) Weight: NR Level:university table tennis player TT:NR	Freq.: 3 times/week, time:NR Length: 9 weeks	E.G.,: Periodized strength training CG: Traditional strength training	Speed: 50-M run (seconds). standing long jump (cm). Endurance:800-m run (minutes) Flexibility: Sit-and-reach (cm) Agility: X-shaped forward and backward run (seconds)	50-M run: 9.35→8.67 Standing long jump: 174.1→191.9, sit-and-reach: 24.09→30.07 cm X-shaped run: 10.23→8.99 s, 800-m run: 3.78→3.20 min
Hao (2023)	RCT	Sport college young table tennis players	E.G., = 20, CG = 20 Sex = NR Age:19 (YR) Height:169.66 ± 6.05 (CM) Weight: 65.78 ± 5.55 (KG) Level:university table tennis player TT:NR	Freq.: 2 times/week, time:60mins Length: 8 weeks	E.G.,: Lower body strength training CG: Traditional speed movement training	Speed: 30 m run time; standing triple jump distance; 30 s double-swing jump rope; 1 min alternate jumps Endurance: 20 m shuttle run time; 1 min alternate jumps Agility: Low-center-of-gravity four-point run; left–right lateral run; straight-line shuttle run	30-s double-swing rope skipping↑ 20-M shuttle run↑Standing triple jump↑ Low-center-of-gravity four-point running↑ Left and right lateral running↑ 30-M run↑ Actual combat effect↑ Push and block on the right table↑ Full-table non-fixed point swing speed↑
Xiong et al. (2022)	RCT	Chinese elite female table tennis players	E.G., = 12, CG = 12 Sex = famale Age:23.5 ± 1.1 (YR) Height:164.2 ± 4.5 (CM) Weight: 58.7 ± 4.1 (KG) BMI: 23.8 ± 4.1 Level:university table tennis player TT:NR	Freq.: 4 times/week, time:30mins Length: 8 weeks	E.G.,: INT CG: Traditional speed movement training	Strength: 1RM (maximum strength) Vertical jump Agility: Y Test	1RM: ↑ Vertical jump height: ↑ Y Balance test ↑ 30m sprint↔ Y Test left leg front direction↔
Chen Q. et al. (2025)	RCT	Youth table tennis player	E.G., = 15, CG = 15 Sex = male Age:15–17 (YR) Height:167.256 ± 2.83 weight: 56.75 ± 0.66 Level:Provincial team members TT:NR	Freq.: 3 times/week, time:30mins Length: 8 weeks	E.G.,1: Multi-directional movement training combined with balance training E.G.,2: Multi-directional movement training (same as MB group) + stable surface balance training	Agility: (COD) Hexagon agility test T-half change speed test 3-M side slide test A-movement test Y-Balance TestPush-and-block side lunge right test	Hexagonal agility test (Partial η^2^ = 0.361), T-half change of direction test (η^2^ = 0.739), 3m sideways slide test (η^2^ = 0.725), A-move test (η^2^ = 0.791), Y-balance test (dominant leg η^2^ = 0.657, non-dominant leg η^2^ = 0.653), push-and-block side lunge right test (η^2^ = 0.815)
Zhu et al. (2024)	RCT	Young table tennis players at youth training academy	E.G.,1 = 8 E.G.,2 = 8 E.G.,3 = 8 CG = 8 Sex = famale Age:19.4 ± 1.2 (YR) Height:179.4 ± 4.1 weight: 77.8 ± 5.3 BMI: 23.8 ± 4.1 Level:NR TT:5years (YR)	Freq.: 3 times/week, time:80mins Length: 8 weeks	E.G.,1: PT E.G.,2: RT E.G.,3: CS CG: Traditional group	Strength: 3 kg medicine ball sitting chest throw (cm). Bench press 1RM (kg). Sprrd: Visual stimulus button reaction (ms) Endurance: 30-s upper limb Wingate test (peak power, average power, W)	Muscle power (medicine ball throw↑(CS-PT/TS-PT) Maximum strength (bench press 1RM)↑ (CS-RT/TS-RT) Aerobic power (Wingate test)↑PT ES = 1.66–1.83 (large) higher than the RT group Reaction time↑
Dong et al. (2025)	RCT	College table tennis players	E.G., = 9 CG = 9 Sex:11M, 7F Age: 20.22 ± 1.09 (YR) Weight: 64.56 ± 9.91 Height: 172.67 ± 8.25 Level: Participated in national competitions TT:13.33 ± 2.06	Freq.: 3 times/week, time:NR Length: 12 weeks	E.G.,: Periodized core training CG: Continuous training	Muscle strength: Grip, CMJ, SLJ. Muscular endurance: Bridge L, bridge R, Plank Speed: 30 m sprint Agility: Edgren sidestep Anaerobic capacity: 400m	Muscular endurance: Bridge L↑, bridge R↑, Plank↑ Speed: 30 m sprint↑ Agility: Edgren sidestep↑ Anaerobic capacity: 400m↑
Wu et al. (2023)	RCT	Freshmen of table tennis specialty in a university	E.G., = 20 CG = 20 Sex = NR Age:18–20(YR) Height:165.87 ± 6.64 Weight:58.71 ± 5.82 Level:University team TT: NR	Freq.: 3 times/week, time:1h Length: 6 weeks	E.G.,:Functional training CG group: Regular competitive training only	50 m run, standing long jump, 1-min sit up, 1-min jump rope, 2.74-m side step	2.74-M side step↑, standing long jump↑

E.G., experimental group; CG, control group; WT, weight; NR, notreported; HR: heart rate; HT, height; Freq.,frequency; M, Male;F, female; YR, year; ↑, signifificant within-group improvement from pretest to post-test; ↔, non-signifificant within-group change from pretest to post-test;VO_2_max, Maximal Oxygen Uptake; ES, effect size; RM, repetition maximum; CMJ, countermovement jump; SJ, squat jump; DJ, drop jump; HRmax, Maximum Heart Rate; LA, blood lactate concentration; TTR, table tennis rating; YBT, Y-Balance Test; HIMT, High-Intensity Interval Multi-ball Training; PT, plyometric training; RT, resistance training; INT, integrated neuromuscular training; CS, cluster set; TS, traditional set; η^2^, Eta-squared; RSI, reactive strength index SBJ, standing broad jump; BAH, balancing on alternate heels; VPA, vertical power assessment; HGR, hand grip resistance; Training Time, TT.

### 3.4 Intervention characteristics

The studies included in this review utilized 10 distinct intervention protocols (Table 3). These interventions included Progressive Strength Training (Zhang, 2022), Lower Limb Flexibility Training (Wang and Zhou, 2023), Upper Limb Strength Resistance Training (Dehua, 2023), Periodized Strength Training (Meng, 2023), Lower Body Strength Training (Hao, 2023), Integrated Neuromuscular Training (Xiong et al., 2022), Multi-directional Movement Training (Chen Q. et al., 2025), Plyometric Training, Resistance Training, Cluster Set, and Traditional Set (Zhu et al., 2024), Periodized Core Training (Dong et al., 2025), and Functional Training (Wu et al., 2023). The length of the interventions across the 10 studies ranged from 6 to 12 weeks, with an average of 8.4 weeks. Most studies implemented training 2 to 4 times per week, with session durations ranging from 60 to 80 min. However, one study (Wang and Zhou, 2023) did not report training frequency; with this exception, all studies reported the specific training duration. All included studies employed a randomized controlled design with a pre–post intervention structure (Dong et al., 2025; Hao, 2023; Wang and Zhou, 2023; Xiong et al., 2022; Zhu et al., 2024; Zhang, 2022; Dehua, 2023; Meng, 2023; Chen Q. et al., 2025; Wu et al., 2023). Regarding group allocation, nine studies included one experimental group and one control group (Dong et al., 2025; Hao, 2023; Wang and Zhou, 2023; Xiong et al., 2022; Dehua, 2023; Meng, 2023; Chen Q. et al., 2025; Wu et al., 2023), while one study included multiple experimental groups and a control group to compare different training methods (Zhu et al., 2024).

### 3.5 Outcome

In this study, the results were categorized according to table tennis players’ performance in speed, strength, endurance, flexibility, and agility. For this purpose, each author independently categorized the included studies based on the specific domains examined. Any disagreements that arose during this process were thoroughly discussed among all authors until a unanimous consensus was reached. Subsequently, the findings of the 10 studies were systematically compiled, summarized, and analyzed.

#### 3.5.1 Effect of exercise training on speed performance in youth table tennis players

Among the 10 studies included in this systematic review, seven investigated the effects of exercise training interventions on speed-related performance in table tennis players, drawing on participants from diverse athletic levels such as provincial or professional teams (Chen Q. et al., 2025), sports school students (Wang and Zhou, 2023), and university team athletes (Dong et al., 2025; Hao, 2023; Xiong et al., 2022; Meng, 2023; Wu et al., 2023). Collectively, these studies encompassed 60 participants aged between 15 and 23 years (Dong et al., 2025; Hao, 2023; Wang and Zhou, 2023; Xiong et al., 2022; Meng, 2023; Chen Q. et al., 2025; Wu et al., 2023). A variety of speed assessment protocols were employed, including the 30-m sprint (Hao, 2023; Wang and Zhou, 2023; Xiong et al., 2022), 50-m sprint (Meng, 2023; Wu et al., 2023), 30-m and 400-m sprints (Dong et al., 2025), low-center-of-gravity four-point running time and left-right lateral running time (Hao, 2023), and the 2.74-m side step (Wu et al., 2023).

Of the two studies utilizing the 30-m sprint as a primary outcome measure, two reported statistically significant improvements following intervention (Hao, 2023; Wang and Zhou, 2023), while one study reported non-significant findings (Xiong et al., 2022). Specifically, in the experimental groups, 30-m sprint time decreased by approximately 0.40 s (from 4.55 to 4.15 s), compared with a reduction of 0.22 s in the corresponding control groups (from 4.58 to 4.36 s). However, the between-group difference after the intervention was not statistically significant (P > 0.05). In contrast, Meng (2023) reported a significant between-group difference in 50-m sprint performance, with running time decreasing from 9.35 to 8.67 s in the experimental group (P < 0.05). The experimental group demonstrated faster sprint times than the control group at post-test (8.67 s vs. 9.14 s, P < 0.05), indicating that the intervention was more effective in enhancing sprint speed compared with the control condition (Meng, 2023). However, another study (Wu et al., 2023) showed that although the experimental group improved in the 50-m test, reducing time by 0.33 s, the between-group difference was not significant.

One study (Hao, 2023) reported that running time in the experimental group significantly decreased from 39.1 to 37.0 s for the four-point running test and from 13.98 to 11.95 s for the lateral running test (P < 0.01). Between-group comparisons at post-test revealed that the experimental group demonstrated significantly faster running times than the control group for both tests, indicating a superior training effect. In contrast, the control group showed only minimal and non-significant changes (Hao, 2023).

Chen Q. et al. (2025) reported significant between-group differences at post-test in both the 3-m sideways slide and A-type movement tests, with the experimental group demonstrating superior lateral movement performance compared with the control group (P < 0.05). At post-test, the experimental group achieved a substantially shorter completion time in the 3-m sideways slide (7.38 ± 0.43 s) than the control group (7.78 ± 0.60 s), corresponding to a large effect size (η^2^ = 0.725). Within-group analyses further showed significant pre–post improvements in the experimental group (from 8.23 ± 0.41 s to 7.38 ± 0.43 s), whereas the control group exhibited only small and non-significant changes.

Wu et al. (2023) reported a significant inter-group difference in the 2.74-m side step at post-test, with the intervention group performing better than the control group (P < 0.01). However, one study (Dong et al., 2025) found no significant between-group differences in the 30-m or 400-m sprints. Within-group comparisons revealed significant improvements from pre-test to post-test in both the 30-m and 400-m tests for the experimental group (P < 0.01).

#### 3.5.2 Effect of exercise training on strength performance in youth table tennis players

Among the 10 studies included in this systematic review, nine focused on evaluating the effects of exercise training on strength-related performance in table tennis players. Collectively, these studies involved a total of 248 athletes aged between 15 and 24 years (Hao, 2023; Wang and Zhou, 2023; Xiong et al., 2022; Zhu et al., 2024; Zhang, 2022; Dehua, 2023; Meng, 2023; Chen Q. et al., 2025; Wu et al., 2023). The studies encompassed a wide range of athlete levels, including students from specialized sports schools (Wang and Zhou, 2023), university-level table tennis athletes (Hao, 2023; Xiong et al., 2022; Zhang, 2022; Dehua, 2023; Meng, 2023; Wu et al., 2023), provincial team members (Chen Q. et al., 2025), and athletes with more than 5 years of training experience, although their competitive level was not specified (Zhu et al., 2024).

A variety of strength-related assessments were employed across the included studies, such as the one-repetition maximum (1RM) squat (Wang and Zhou, 2023; Xiong et al., 2022; Zhu et al., 2024), countermovement jump (CMJ) height (Wang and Zhou, 2023; Xiong et al., 2022), standing long jump (Meng, 2023; Wu et al., 2023), triple jump (Hao, 2023), and shoulder/elbow/wrist joint flexion peak torque (Zhang, 2022).

Two studies assessed 1RM squat performance and consistently reported statistically significant improvements following the intervention (Wang and Zhou, 2023; Xiong et al., 2022). One study found that the experimental group showed significant increases in 1RM squat strength (from 79.26 ± 8.43 kg to 79.65 ± 9.59 kg, P = 0.027) and CMJ height (from 34.55 ± 5.15 cm to 34.92 ± 4.75 cm, P = 0.016), whereas no meaningful changes were observed in the control group (Wang and Zhou, 2023). Another study reported a significant between-group advantage in post-intervention posterior-chain performance in favor of the experimental group (P < 0.001) (Xiong et al., 2022). Similarly, significant post-test between-group differences in vertical jump performance were observed, with the experimental groups exhibiting greater improvements compared with the control groups (P < 0.05) (Wang and Zhou, 2023).

Standing long jump performance improved significantly in two studies (Meng, 2023; Wu et al., 2023), with one reporting a between-group difference of 2.71 cm (P = 0.048) in favor of the experimental group (Wu et al., 2023). Triple jump distance also showed significant gains (Hao, 2023), increasing by 0.13 m (from 5.38 m to 5.51 m, P < 0.01). In addition, one study reported significant between-group differences in peak torque of the elbow and wrist joints at post-test. The experimental group demonstrated significant improvements in both maximal strength (60°/s) and explosive force (450°/s) during elbow flexion and extension, as well as in shoulder flexor explosive power. In contrast, the control group showed no significant improvements in any of the indicators (Zhang, 2022).

Two studies evaluated sport-specific strength indicators, including grip strength (Dehua, 2023) and medicine ball throw (Zhu et al., 2024). A study using a handheld dynamometer to assess grip strength showed that within the experimental group, grip strength significantly increased from 43.15 kg to 47.17 kg (P < 0.05). Although both groups showed improvement, the increase in the experimental group was statistically significant, while the control group showed only a non-significant increase (from 43.10 kg to 46.87 kg, P > 0.05) (Dehua, 2023). Medicine ball throwing performance, reflecting upper-limb explosiveness, was significantly enhanced in the plyometric training groups, with a large effect size observed in both the cluster-set plyometric training group (CS-PT, ES = 1.21) and the traditional-set plyometric training group (TS-PT, ES = 1.02). Between-group comparisons further showed that both plyometric training configurations exhibited significantly greater improvements than their respective resistance training counterparts (CS-PT vs. CS-RT, P = 0.021; TS-PT vs. TS-RT, P = 0.018) (Zhu et al., 2024).

#### 3.5.3 Effect of exercise training on endurance performance in youth table tennis players

Endurance in sports training generally refers to the ability to sustain physical activity, including aerobic endurance, anaerobic capacity, and muscular endurance, which together determine an athlete’s capacity to maintain performance during prolonged or repeated efforts (Biernat et al., 2018). Among the 10 studies included in this systematic review, four specifically evaluated the effects of exercise training on endurance in table tennis players. These studies collectively involved 108 athletes aged between 18 and 24 years (Dong et al., 2025; Hao, 2023; Wang and Zhou, 2023; Meng, 2023), encompassing students from sports schools and university-level table tennis players (Dong et al., 2025; Hao, 2023; Wang and Zhou, 2023; Meng, 2023).

Endurance assessments in these studies reflected three domains of endurance capacity: aerobic endurance (e.g., 800-m run (Meng, 2023)), muscular endurance (e.g., Bridge L, Bridge R, Plank (Dong et al., 2025)), and anaerobic endurance (e.g., 30-s double-under and 1-min alternating jump tests (Hao, 2023)).

One study incorporated the 800-m run as an aerobic endurance indicator. Results demonstrated a significant improvement in the experimental group’s 800-m run time, reduced from 4 min 18 s to 3 min 20 s (P < 0.05). Between-group comparisons at post-intervention further indicated significantly faster times in the experimental group compared with the control group (P < 0.05). Although the control group also showed a modest improvement (from 3.97 ± 0.35 min to 3.71 ± 0.35 min, P = 0.032) (Meng, 2023).

In the anaerobic endurance domain, the experimental group achieved significantly higher repetition counts in both the 30-s double-under test and the 1-min alternate jumping test, with between-group differences favoring the experimental group at post-intervention (P < 0.05). Specifically, in the 30-s double-under test, the experimental group improved from 56.53 ± 3.33 to 61.88 ± 2.46 (P < 0.05), whereas the control group showed a smaller increase from 57.52 ± 2.67 to 59.36 ± 1.76 (P < 0.05) (Hao, 2023).

For muscular endurance, one study (Wang and Zhou, 2023) reported improvements in 1-min squat performance. The experimental group increased repetitions from 51.92 ± 6.43 to 52.88 ± 5.46 (P = 0.005), while the control group showed virtually no change (52.47 ± 5.88 to 52.41 ± 5.80, P = 0.743). Another study (Dong et al., 2025) assessed muscular endurance via Bridge L, Bridge R, and Plank tests. Significant group × time interactions were observed for all three measures: Bridge L (F = 5.329, P < 0.05, ηp^2^ = 0.25), Bridge R (F = 13.351, P < 0.001, ηp^2^ = 0.455), and Plank (F = 9.052, P < 0.01, ηp^2^ = 0.361), indicating superior improvements in the experimental group compared with the control group.

#### 3.5.4 Effect of exercise training on flexibility performance in youth table tennis players

Among the 10 studies included in this systematic review, five specifically evaluated the effects of exercise training on flexibility in table tennis players. These studies involved a total of 136 athletes aged between 15 and 24 years (Wang and Zhou, 2023; Xiong et al., 2022; Zhu et al., 2024; Meng, 2023; Chen Q. et al., 2025). Flexibility assessments primarily included measurements of joint range of motion, such as seated forward bend distance (Wang and Zhou, 2023; Meng, 2023), hip flexion/extension angle, ankle dorsiflexion angle (Wang and Zhou, 2023), and ankle flexibility during various jump movements (Xiong et al., 2022; Zhu et al., 2024).

Two studies reported significant improvements in seated forward bend distance following the intervention (Wang and Zhou, 2023; Meng, 2023). In addition, Wang and Zhou (Wang and Zhou, 2023) observed statistically significant increases in both hip flexion/extension and ankle dorsiflexion angles after the training period. For example, ankle dorsiflexion improved markedly in the experimental group (from 16.57° to 20.33°, P = 0.007) but remained essentially unchanged in the control group (from 16.31° to 16.86°, P = 0.568), highlighting the superior flexibility gains induced by the intervention. Similarly, hip flexion angle increased from 101.47° ± 11.89° to 113.92° ± 10.74° (P < 0.05) in the experimental group, whereas the control group showed a slight decline (from 111.20° ± 11.02° to 110.13° ± 9.46°, P > 0.05). Seated forward bend distance also increased significantly in the experimental group (from 24.09 ± 6.72 cm to 30.07 ± 7.18 cm, P < 0.05), while no meaningful change was observed in the control group (from 25.28 ± 8.69 cm to 25.65 ± 6.66 cm, P > 0.05) (Wang and Zhou, 2023).

Furthermore, several studies reported improved ankle mobility in the experimental group, suggesting that enhanced joint flexibility supports postural control and functional stability (Xiong et al., 2022; Zhu et al., 2024; Chen Q. et al., 2025). Overall, most flexibility-related indicators showed statistically significant improvements in the experimental groups from pre-to post-intervention, whereas no such improvements were observed in the control groups, indicating a clear between-group advantage of the interventions.

#### 3.5.5 Effect of exercise training on agility performance in youth table tennis players

Among the 10 studies included in this systematic review, five examined the effects of exercise training on agility in table tennis players, involving a total of 138 athletes aged 15–24 years (Dong et al., 2025; Hao, 2023; Wang and Zhou, 2023; Meng, 2023; Chen Q. et al., 2025). Agility performance was primarily assessed through change-of-direction (COD) tests, including the T-test, Illinois agility test, figure-eight running, and X-pattern shuttle runs (Meng, 2023; Chen Q. et al., 2025). Multidirectional agility was evaluated using the hexagon agility test and cross-quadrant jump tests (Wang and Zhou, 2023; Chen Q. et al., 2025). Several studies also incorporated table tennis–specific agility measures, such as fixed-point stroke frequency and full-table random stroke frequency (Wang and Zhou, 2023; Chen Q. et al., 2025), as well as the Edgren side-step test (Dong et al., 2025). Additional complementary tests included the 30-s double-under jump rope test and the 20-m shuttle run, which reflect elements of coordination and agility (Hao, 2023).

The intervention group demonstrated significantly superior performance compared with the control group across multiple agility tests. For example, in the figure-eight running test, Illinois agility test, and T-test, the experimental group consistently exhibited better performance than the control group (Chen Q. et al., 2025). In the figure-eight running test, the intervention group achieved a 0.174-s shorter completion time than the control group (P < 0.05). Post-intervention comparisons further confirmed that agility performance in the experimental group was significantly superior to that of the control group (Chen Q. et al., 2025). In addition, two studies reported significant within-group improvements, with marked reductions in X-pattern shuttle run time (Meng, 2023) and 20-m shuttle run time (Hao, 2023). Specifically, X-pattern shuttle run time decreased from 10.23 s to 8.99 s (P < 0.05) (Meng, 2023), while 20-m shuttle run time was significantly reduced from 22.9 s to 19.6 s (P < 0.01) (Hao, 2023). Similarly, the Edgren side-step test showed significant between-group differences at post-test (F = 12.487, P < 0.05) (Dong et al., 2025).

For within-group comparisons, one study (Wang and Zhou, 2023) reported significant improvements in cross-quadrant jumping performance in the experimental group. After the intervention, subjects also demonstrated significant gains in side-slide repetitions. The improvement in 3-m sideslip performance was more pronounced in the experimental group (from 26.66 ± 1.23 to 28.16 ± 1.33, P = 0.025) compared with the control group (from 26.70 ± 1.30 to 27.36 ± 1.23, P = 0.070), indicating a clearer training advantage (Wang and Zhou, 2023).

## 4 Discussion

This study aims to investigate the effects of exercise training interventions on the physical fitness of young table tennis players and to evaluate their potential benefits. Preliminary evidence from the 10 included studies indicates that exercise training positively contributes to improvements in speed, agility, strength, endurance, and flexibility, thereby enhancing athletes’ overall physical fitness. Although the findings suggest that exercise training is beneficial, variability in study quality underscores the need for more rigorous research in the future.

### 4.1 Effect of exercise training on speed performance in youth table tennis players

The beneficial effects of exercise training on the speed of table tennis players have been consistently highlighted in the existing literature. Several studies have provided robust evidence, with most speed assessments focusing on running performance (Dong et al., 2025; Hao, 2023; Wang and Zhou, 2023; Xiong et al., 2022; Meng, 2023; Chen Q. et al., 2025; Wu et al., 2023). Among the commonly used indicators, straight-line sprint ability over 30 and 50 m has shown notable improvements (Dong et al., 2025; Hao, 2023; Wang and Zhou, 2023; Xiong et al., 2022; Meng, 2023). For instance, following lower-body flexibility training, 30-m sprint time decreased by 0.40 s (from 4.55 s to 4.15 s) (Wang and Zhou, 2023). This improvement is particularly relevant for table tennis, as it reflects enhanced capacity to recover quickly from wide-angle returns to the table, typically covering a distance of approximately 3 m. This finding aligns with prior studies reporting that targeted lower-limb flexibility or plyometric programs can produce comparable reductions in sprint time among youth athletes, typically ranging from 0.20 to 0.45 s, suggesting that neuromuscular adaptations may occur across different training modalities (Başkaya et al., 2024). Such improvements play a pivotal role in enhancing an athlete’s responsiveness during extended rallies, particularly in scenarios requiring rapid recovery to the forehand side after a wide-angle return (Kim and Kim, 2013; Taş, 2017). The underlying mechanisms are thought to involve enhanced stretch–shortening cycle efficiency of the lower limbs and improved recruitment of type II muscle fibers, both of which accelerate force production during explosive starts and transitions (Taş, 2017).

Similarly, following a periodized strength training intervention, 50-m sprint time decreased from 9.35 s to 8.67 s (Meng, 2023), reflecting enhanced neuromuscular efficiency and improved explosive acceleration capacity of the lower limbs—adaptations that are more directly linked to sprint performance in table tennis (Smirniotou et al., 2008). These improvements further support the notion that speed-related gains in young athletes stem primarily from increases in force production, motor unit recruitment, and movement coordination rather than metabolic changes (Young et al., 1995).

In contrast, two studies reported no statistically significant between-group differences in 30-m and 50-m sprint performance when compared with control conditions (Dong et al., 2025; Wu et al., 2023). However, within-group analyses in these studies revealed significant improvements over time. Specifically, one study demonstrated significant reductions in 30-m sprint time from pre-to mid-test and from mid-to post-test (P < 0.01) (Dong et al., 2025). Similarly, another study reported a significant within-group improvement in 50-m sprint performance, with sprint time reduced by 0.55 s following the intervention (Wu et al., 2023).

This discrepancy may be attributed to the nature of the training interventions. For example, core training programs primarily target trunk stability and neuromuscular control rather than directly enhancing lower-limb force production capacity (Hibb et al., 2008; Chen B. et al., 2025). Likewise, certain functional training protocols may not provide sufficient overload stimulus or sprint-specific neuromuscular adaptations to elicit statistically superior improvements compared with routine table tennis training (Bustos Carvajal and Arias Coronel, 2025). In contrast, exercise programs that specifically emphasize lower-limb power development, sprint mechanics, or longer intervention duration appear more likely to produce measurable between-group advantages in sprint performance (Chen B. et al., 2025).

Collectively, these findings suggest that improvements in sprint ability among young table tennis players may be more pronounced when training interventions directly target lower-limb explosive strength and are implemented with adequate intensity and specificity.

Another important aspect of current research concerns multidirectional speed, particularly change-of-direction (COD) speed, as reflected in low-center-of-gravity four-point running tests (Hao, 2023). These assessments are designed to simulate the rapid transitional movements required in table tennis, such as fast shifts between forehand and backhand positions at the table (Wong et al., 2020a). For instance, during serve reception, explosive force generation is required when the knee flexion angle reaches 110° or less, following rapid rotation and weight transfer from the backhand side. This movement pattern closely mirrors the practical demands of the “Z-shaped” footwork sequences commonly observed in competitive play (Chen Q. et al., 2025). The effectiveness of these turning and transition-speed actions is largely attributed to enhanced neuromuscular coordination between the trunk and lower limbs, which enables trunk stabilization while allowing rapid redirection of force and minimized transition time during sharp positional shifts (Wong et al., 2020a; Fuchs and Lames, 2021). Similar findings have been reported in other movement-analysis studies, emphasizing that improvements in COD speed among youth racket-sport athletes are strongly mediated by enhanced trunk stability and lower-limb neuromuscular control (Taş, 2017). In line with this, plyometric-based interventions have demonstrated concurrent improvements in squat jump, countermovement jump, and COD performance, suggesting that enhancements in reactive strength and neuromuscular control may translate into faster directional transitions even in the absence of maximal sprint gains (Chen Q. et al., 2025). Collectively, these studies suggest that athletes with better proximal stability exhibit shorter transition times and faster execution of forehand–backhand movements, providing cross-study support for the speed-related mechanisms observed in the reviewed table tennis research.

These findings are consistent with those reported in badminton and tennis, where directional change execution speed in male university athletes improved significantly following twice-weekly training interventions (P < 0.05) (Stovba et al., 2020; Gussanto, 2025). However, unlike badminton and tennis, table tennis performance relies predominantly on rapid, high-frequency movements executed within a very short displacement range of approximately 2–4 m (Stovba et al., 2020). This contextual difference may explain why enhancing short-distance COD speed is more critical for table tennis players than improving absolute linear sprint velocity (Phomsoupha et al., 2018). Mechanistically, these brief explosive movement sequences depend heavily on rapid neuromuscular activation and efficient lower-limb force transmission, allowing athletes to complete repeated acceleration–deceleration cycles with minimal transition time under highly constrained spatial conditions (Sybil et al., 2025; Gussanto, 2025). The underlying adaptations are primarily attributed to lower-limb–specific neuromuscular improvements. Interventions included 1RM squats, countermovement jumps, and weighted squat jumps, which enhanced type II muscle fiber recruitment efficiency and lower-limb explosive power, thereby supporting faster movement execution during directional transitions (Hao, 2023; Wang and Zhou, 2023; Xiong et al., 2022; Meng, 2023; Chen Q. et al., 2025).

While these findings collectively suggest a beneficial role of exercise training in enhancing speed-related performance in table tennis, future research could extend beyond current protocols. Specifically, further studies are warranted to examine whether similar adaptations occur across different age groups, competitive levels, and between male and female athletes. Moreover, future investigations could incorporate more integrative testing systems that go beyond isolated linear sprint or single-task directional movement speed assessments, and instead include table tennis–specific movement sequences such as stroke initiation, rally recovery, and rapid multi-directional footwork patterns. Such approaches would provide a more comprehensive evaluation of how speed-oriented physical conditioning translates into effective technical execution and, ultimately, competitive performance in table tennis.

### 4.2 Effect of exercise training on strength performance in youth table tennis players

The accumulated findings on the beneficial effects of exercise training on the strength of table tennis players have provided substantial evidence in this field (Hao, 2023; Wang and Zhou, 2023; Xiong et al., 2022; Zhu et al., 2024; Zhang, 2022; Dehua, 2023; Meng, 2023; Chen Q. et al., 2025; Wu et al., 2023). Increasing evidence confirms that exercise interventions can significantly enhance athletes’ strength, particularly in lower-limb maximal strength and explosive power. For example, studies have shown that both maximal strength and explosive power of the lower limbs can be improved simultaneously through progressive load squat training and combined dynamic and static training (Hao, 2023; Wang and Zhou, 2023). Furthermore, several independent studies reported significant increases in 1RM squat performance following structured resistance training (Hao, 2023; Wang and Zhou, 2023; Meng, 2023). It is worth noting that Xiong et al. (2022) observed an 11.61% increase in 1RM and a 15.4% increase in CMJ height in the experimental group following integrated neuromuscular training (INT), both statistically significant (P < 0.001). These enhancements are likely the result of neuromuscular adaptations. Periodic resistance training, typically conducted three times per week at an intensity ranging from 70% to 85% of 1RM, has been shown to improve motor unit synchronization and enhance type II muscle fiber recruitment efficiency (Zhu et al., 2024).

Similar training mechanisms have also been observed in other sports where explosive power plays a central role. For example, in badminton-specific studies, periodic resistance training led to marked improvements in lower limb strength and vertical jump performance, contributing to enhanced court movement efficiency and rapid movement capacity (Lin et al., 2025; Sonoda et al., 2018; He et al., 2022). Likewise, in the context of tennis, an 8-week program combining heavy strength training with explosive power cycles resulted in a 13% increase in 1RM squat and a 10% improvement in the reactive strength index (RSI), reflecting a comparable trajectory of neuromuscular adaptation (Deng et al., 2022). The improvement in CMJ height is directly related to optimization of the force-velocity curve during the propulsion phase, which is critical for ground reaction force output during rapid starts and strokes (Wang and Zhou, 2023). Another study found that athletes’ triple jump distance increased by 0.13 m following lower body strength training (Hao, 2023).

Such improvements reflect enhanced synergistic force production in the hip-knee-ankle kinetic chain, which may be attributed to stretch reflex activation induced by compound training (e.g., box jumps combined with multi-directional sprints) (Chen Q. et al., 2025). For example, one study reported that a 6-week functional training program significantly improved standing long jump performance in young table tennis players (Wu et al., 2023). This may be explained by the ability of functional training to enhance neuromuscular coordination, intermuscular synchronization, and force transmission efficiency through multi-joint, movement-integrated exercises (Wu et al., 2023). Similar findings have been reported in other sports, where functional training interventions have been shown to improve lower-limb explosive performance and movement efficiency in disciplines such as basketball and soccer (Xiao et al., 2025).

Furthermore, Zhang (2022) reported significant increases in peak flexion torque of the elbow joint at 60°/s and 450°/s, peak extension torque at 450°/s, and peak extension torque of the wrist joint at both 60°/s and 450°/s, reflecting enhanced upper-limb isokinetic strength following the intervention. These improvements are closely associated with stroke production in table tennis, as elbow and wrist torque generation plays a critical role in accelerating the racket during topspin execution and rapid directional changes (Wong et al., 2020b). The observed increases in joint-specific torque capacity therefore suggest enhanced force transmission efficiency and explosive power during high-velocity technical actions (Bańkosz and Winiarski, 2018).

In terms of upper-body strength, existing studies have shown that both plyometric training (PT) and resistance training (RT) produce substantial improvements in medicine ball throwing performance among young table tennis players, with large effect sizes (e.g., ES = 1.21) (Zhu et al., 2024). These performance gains are likely attributed to enhanced neuromuscular recruitment efficiency and improved coordination between the upper limbs and core musculature, which together promote more effective force transmission during explosive actions (Lin et al., 2025). Similar upper-limb adaptive training effects have also been reported in badminton. Through the combined application of plyometric and resistance training, athletes in this sport achieve significant improvements in overhead throwing performance and racket-head speed. Research on adolescent badminton players has shown that a combined plyometric–resistance training program increased two-hand medicine-ball chest-throw distance from 5.4 ± 0.5 m to 6.2 ± 0.6 m (P < 0.01), an improvement that closely parallels the gains in upper-limb explosive power observed in medicine-ball-throwing studies among table tennis athletes (Liu et al., 2024). PT facilitates rapid activation of type II muscle fibers through high-intensity stretch–shortening contractions, whereas RT increases muscle cross-sectional area and strength reserves through progressive overload; the combination of these mechanisms contributes to greater upper-limb explosive power and improved throwing performance (Zhu et al., 2024).

Additionally, research has shown that exercise-based interventions lead to significant improvements in grip strength performance in young table tennis athletes (Dong et al., 2025; Dehua, 2023). Specifically, one study (Dong et al., 2025) observed significant within-group gains in grip strength following a 12-week periodized core training program, but no significant between-group differences were found. In contrast, an 8-week resistance training program (Dehua, 2023) demonstrated significant between-group improvements. This discrepancy highlights an important principle: different training modalities target distinct physiological adaptations. While core training primarily enhances trunk stability and neuromuscular control, resistance training provides the specific overload necessary for rapid gains in maximal strength and hypertrophy (Tillin and Folland, 2014).

Therefore, future research should adopt a comprehensive testing program that integrates upper and lower limb indicators and table tennis–specific strength performance, and conduct more comparative studies to clarify how different strength training methods translate into sport-specific advantages.

### 4.3 Effect of exercise training on endurance performance in youth table tennis players

Substantial evidence has been established regarding the beneficial effects of exercise training on the endurance of table tennis players. The underlying mechanisms involve the synergistic enhancement of aerobic capacity, anaerobic metabolic efficiency, and muscular endurance (Dong et al., 2025; Hao, 2023; Wang and Zhou, 2023; Meng, 2023). These endurance improvements correspond closely to the physiological characteristics required in competitive table tennis. Unlike many continuous endurance sports, table tennis relies on a unique blend of aerobic endurance to sustain performance over matches lasting 40–60 min, and anaerobic endurance to repeatedly generate short bursts of high-intensity actions. During play, each rally typically lasts 3–5 s and involves rapid accelerations, explosive strokes, and frequent direction changes, followed by brief recovery periods of 10–20 s (Fuchs et al., 2018; Pradas de la Fuente et al., 2013).

In terms of aerobic endurance, studies have confirmed the effectiveness of periodized strength training on aerobic capacity. Following a periodized strength training intervention, 800-m run time significantly decreased from 3.78 min to 3.20 min (Meng, 2023), which is equivalent to an approximate 12% increase in relative oxygen uptake. This improvement may be attributed to increased mitochondrial density and capillary surface area within muscle fibers, thereby enhancing oxygen transport and utilization efficiency (Figueiredo, 2019). Similarly, another study found that a mixed endurance program combining circuit running and dynamic resistance training significantly improved VO_2_max and heart rate recovery within 8 weeks, further supporting the transferable benefits of interdisciplinary endurance training for table tennis players (Taipale et al., 2013; Sugiyanto et al., 2024). The shared mechanism underlying these improvements lies in the dual adaptations of cardiopulmonary function and peripheral metabolism, manifested as enhanced oxygen supply efficiency and recovery capacity (Pradas de la Fuente et al., 2013).

Research shows that lower-body strength training substantially enhances short-duration anaerobic endurance in young table tennis athletes. For example, the number of double-under jumps increased from 56.5 to 61.9 repetitions in 30 s (P < 0.05), while alternating jumps in 1 minute also improved significantly (P < 0.05) (Hao, 2023). These changes reflect enhanced creatine phosphate resynthesis capacity and improved fatigue resistance of type IIb muscle fibers. Similar improvements have been observed in adolescent badminton and tennis players, where repeated high-intensity footwork drills significantly improved short-burst jumping and shuttle-run performance, indicating comparable anaerobic adaptations across racket sports (Paoli and Häkkinen, 2025; Ozmen and Aydogmus, 2016).

In addition to anaerobic endurance, lower-limb flexibility training has been shown to improve muscular endurance, with the number of squats completed in 1 minute increasing from 52.88 to 64.05 repetitions—a 21% enhancement (Wang and Zhou, 2023). These adaptations contribute to reduced metabolic cost of movement, allowing young athletes to sustain high-intensity exchanges for longer durations. From a theoretical perspective, repeated flexibility and strength stimuli may enhance muscle fiber recruitment efficiency, improve neuromuscular coordination, and optimize the elastic properties of the lower-limb musculature, collectively contributing to greater endurance and movement economy during rapid, repetitive actions (Bangsbo et al., 2025; Aoki et al., 2025).

Regarding muscular endurance, Dong et al. (2025) demonstrated that a 12-week core training program led to significant between-group improvements in Bridge Left, Bridge Right, and Plank performance (Dong et al., 2025). These findings suggest that targeted core training enhances muscular endurance by improving neuromuscular coordination, stabilizing the trunk, and increasing the efficiency of force transmission across the kinetic chain (Hibb et al., 2008; Ozmen and Aydogmus, 2016). According to the Training Adaptation and Neuromuscular Control Framework, repeated core activation promotes motor unit recruitment and synchronization, thereby enabling athletes to sustain high-intensity postural and movement demands for longer durations (Bustos Carvajal and Arias Coronel, 2025; Ozmen and Aydogmus, 2016).

Similar phenomena of improved muscle endurance have also been observed in sports such as volleyball and basketball (Bal et al., 2011; Soylu and Altundag, 2024). Athletes’ repetitive jumping performance and lower-limb muscular endurance are significantly enhanced through targeted strength training (Cao et al., 2024; Lidor and Ziv, 2010). These consistent findings suggest a common underlying mechanism across different sports, in which enhanced neuromuscular coordination, more efficient muscle fiber recruitment, improved movement economy, and contributions from aerobic energy support collectively delay fatigue and help athletes sustain high-intensity actions over both short and moderately prolonged durations.

### 4.4 Effect of exercise training on flexibility performance in youth table tennis players

A growing body of research has demonstrated that exercise training can significantly enhance flexibility performance in table tennis players, with improvements observed in joint range of motion and the coordination of sport-specific movement chains (Fan et al., 2024). These gains are achieved through improvements in static flexibility, joint mobility, dynamic postural control, and sport-specific movement adaptability, all of which help enhance movement economy and reduce injury risk (Wang and Zhou, 2023). Several exercises included in the intervention may have contributed to these flexibility improvements. The back bridge promotes increased hip-extension range and thoracic lumbar spine mobility by lengthening the anterior kinetic chain, including the rectus femoris, iliopsoas, and abdominal fascia, thereby enhancing the functional extensibility of the trunk and hip joints (Wang and Zhou, 2023; Meng, 2023). This mechanism is supported by intervention designs that incorporated bridge-based or posterior-chain extension exercises and reported significant improvements in hip extension and sit-and-reach performance in the experimental group (P < 0.05) (Wang and Zhou, 2023; Meng, 2023). Sit-ups with trunk rotation repeatedly activate the oblique musculature and facilitate segmental thoracolumbar rotation, contributing to greater rotational range of motion (Xiong et al., 2022; Chen Q. et al., 2025). Similar trunk-rotation–oriented training protocols have been shown to significantly improve trunk mobility and functional flexibility in youth racket-sport athletes (Xiong et al., 2022; Chen Q. et al., 2025). Similarly, back-extension exercises strengthen the posterior kinetic chain while improving lumbar and hip-extension mobility, enabling the spine to move through a fuller extension range under controlled loading (Wang and Zhou, 2023; Meng, 2023). Collectively, these exercise-induced adaptations provide plausible mechanisms explaining the observed improvements in flexibility.

Another study showed that among 15–17-year-old male provincial-team table tennis athletes, dynamic stretching combined with proprioceptive neuromuscular facilitation (PNF) techniques increased fascial sliding efficiency by 18% (Chen Q. et al., 2025). This improvement is believed to result from reduced adhesions between fascial layers, redistributed myofascial tension, and enhanced shear-gliding capacity of deep soft tissues. Similar findings have been reported elsewhere. For example, improvements in hamstring and lower-back flexibility have been attributed to adaptive remodeling of soft tissues (Hopper et al., 2005), driven by enhanced viscoelasticity of the muscle–tendon unit, more orderly collagen fiber alignment, and improved neuromuscular coordination, which together reduce tissue stiffness and increase joint range of motion (Fan et al., 2024).

Research has demonstrated that exercise training can significantly enhance joint mobility in young table tennis players. For example, one study reported notable improvements in hip flexion (increasing to 113.92° ± 10.74°) and ankle dorsiflexion (rising to 20.33° ± 3.30°) following the intervention (P < 0.05) (Wang and Zhou, 2023), indicating an expanded functional range of motion across the lower-limb kinetic chain. These gains create a critical biomechanical foundation for key sport-specific movements, such as maintaining a low center of gravity during lateral slides with knee flexion angles below 110°, and generating rapid push-off or rotational actions that require ankle dorsiflexion of at least 20° (Wang and Zhou, 2023). Similar findings have been reported in studies using INT and traditional weight-training protocols, both of which significantly improved ankle mobility during jump-landing and push-off tasks (Xiong et al., 2022; Zhu et al., 2024). Enhanced hip and ankle mobility not only accelerates center-of-gravity transitions—such as shifting from a forehand lunge to a backhand recovery—but also increases force-transmission efficiency in the push-off phase, with ankle dorsiflexion beyond 20° contributing to approximately a 9% rise in peak ground reaction force (Xiong et al., 2022; Zhu et al., 2024). Collectively, these adaptations improve movement efficiency during rapid directional changes and may reduce the risk of injuries associated with restricted joint mobility (Fan et al., 2024; Dos’ Santos et al., 2022). Comparable evidence from tennis research indicates that improvements in joint range of motion and flexibility are associated with more efficient stroke mechanics and body positioning during rallies, which aligns with the trends observed in the present review (Jansen et al., 2021a; Jansen et al., 2021b; Sinkovic et al., 2022).

This convergence of evidence suggests that flexibility supports technical execution in racket sports by facilitating optimal joint positioning and smoother kinetic-chain coordination. However, given that table tennis is characterized by shorter swing amplitudes and a higher frequency of micro-adjustments than tennis, the specific flexibility demands may differ between sports. Future research should therefore clarify which flexibility components are most relevant for transferring to table-tennis-specific technical performance.

### 4.5 Effect of exercise training on agility performance in youth table tennis players

Several studies have shown that exercise training enhances multiple dimensions of agility in table tennis players, including improvements in functional agility tests, sport-specific movement chain efficiency, and change-of-direction (COD) speed (Dong et al., 2025; Hao, 2023; Wang and Zhou, 2023; Meng, 2023; Chen Q. et al., 2025). The mechanisms behind these improvements are closely related to neuromuscular adaptation and the enhancement of anticipation and decision-making abilities (Bustos Carvajal and Arias Coronel, 2025). Across the included studies, various agility-related tests such as the cross-quadrant jump, hexagon test, figure-eight step, X-shuttle run, T-test, and shuttle-run performance consistently demonstrated significant improvements following targeted strength or plyometric interventions (Hao, 2023; Meng, 2023; Chen Q. et al., 2025). Specifically, one study reported a significant reduction in Edgren sidestep completion time following a periodized core training intervention, indicating enhanced ability in movement initiation, braking control, and directional change efficiency in young table tennis players (Dong et al., 2025).

Despite differences in testing formats, the underlying mechanisms appear largely convergent. Most improvements are attributed to enhanced neuromuscular coordination and elevated eccentric strength of the hip abductors, which increased by up to 22%–28% in some studies, thereby improving deceleration control during directional changes (Wang and Zhou, 2023; Chen Q. et al., 2025). At the same time, gains in ankle dorsiflexion mobility (increased by 12%–15%) and improved elastic energy storage of the Achilles tendon (increased by 12%) facilitated more efficient ground reaction force utilization and faster transition between braking and propulsive phases (Hao, 2023; Wang and Zhou, 2023). These adaptations collectively reduced movement time across multiple COD tasks. For example, COD time decreased by 18%, hexagon agility time improved significantly, and X-shuttle run time decreased by over 1.2 s in collegiate athletes (Meng, 2023).

This mechanism aligns with findings in soccer players, where RSI strongly correlates with COD performance (r = 0.71), supporting the cross-sport validity of this explanation (Başkaya et al., 2024). Another line of interpretation proposes that enhanced phosphagen system capacity, evidenced by a 14% increase in ATP-PCr resynthesis rate, may contribute more prominently to improvements in short-burst shuttle running and repeated acceleration tasks (Hao, 2023).

Taken together, although individual agility tests differ in their specific design, the observed improvements across studies may be attributed to a combination of enhanced eccentric control, greater ankle mobility, improved reactive strength, and more efficient utilization of the phosphagen energy system. These adaptations likely facilitate faster repositioning of the center of mass and reduce energy loss, thereby supporting more efficient and tactically effective change-of-direction movements in young table tennis players.

## 5 Limitation

This systematic review has several limitations that should be acknowledged. First, the literature search was limited to studies retrieved from Web of Science, PubMed, EBSCOhost, and Google Scholar. Although these databases cover a broad range of sports science and health-related research, relevant studies indexed in other databases or unpublished sources may have been overlooked. Therefore, future reviews may benefit from incorporating additional databases to ensure more comprehensive evidence coverage. Second, the present review focused primarily on physical fitness parameters, including speed, strength, endurance, agility, and flexibility. While these indicators represent core components of athletic performance in table tennis, future research may extend beyond physical fitness to include psychosocial factors, perceptual abilities, and technical skill development, which are also critical for long-term performance enhancement in young table tennis players.

## 6 Conclusion

This systematic review provides a comprehensive assessment of the impact of exercise training on the physical health of young table tennis players. The findings highlight that exercise training, through sport-specific adaptations, significantly enhances key fitness components for table tennis, including speed, strength, endurance, flexibility, and agility. In addition, it is recommended to investigate in greater depth the practical transfer of physical fitness improvements to sport-specific performance in table tennis. Finally, expanding the scope of research to include youth athletes of different ages, genders, and skill levels will not only deepen the understanding of characteristics across age groups but also enhance the generalizability of findings across diverse populations.

## 7 Practical application

In table tennis, exercise training is crucial for enhancing physical fitness components such as strength, speed, endurance, flexibility, and agility. With the adjustment of rules to using larger and slower balls, the importance of these qualities has become even more pronounced. Strength and endurance, in particular, are essential for maintaining stroke speed and power throughout rallies. Exercise training, as a comprehensive method for improving athletic performance, has received increasing attention for its role in enhancing these fitness components. When applied to youth table tennis players, it can be transformed into a competitive advantage by improving sport-specific performance. Incorporating exercise training into the periodized programs of youth table tennis players should involve diverse methods that are closely aligned with the sport’s unique movement patterns, force-generation mechanisms, and stroke trajectories. Coaches are encouraged to adopt multifaceted training approaches rather than relying on single modes, ensuring simultaneous improvements in strength, speed, endurance, flexibility, and agility. With the support of systematic exercise training, the physical fitness and competitive performance of youth table tennis players are expected to continue improving.

## Data Availability

The original contributions presented in the study are included in the article/supplementary material, further inquiries can be directed to the corresponding authors.
